# Short-term cardiovascular and mental health responses to Shinrin-Yoku (forest bathing): a systematic review and meta-analysis

**DOI:** 10.3389/fpsyg.2026.1707829

**Published:** 2026-02-11

**Authors:** Aurélio Matos Andrade, Suzane da Fonseca Durães, Lorena Covem Rosa Franco Netto, André Luiz Dutra Fenner, Marco Aurélio Bilibio Carvalho, Guilherme Franco Netto

**Affiliations:** 1 Fiocruz Brasília, Brasília, Brazil; 2 Fundacao Oswaldo Cruz, Rio de Janeiro, Brazil; 3 Instituto Brazileiro de Ecopsicologia (IBE), Brasília, Brazil

**Keywords:** circulatory system, forest bathing, mental disorders, meta-analysis, nature therapy

## Abstract

Interest in the health benefits of Shinrin-Yoku (forest bathing) has increased in recent years, although evidence regarding its efficacy in preventing and managing circulatory problems and mental disorders remains limited. This study conducted a systematic review with meta-analysis to evaluate the effects of forest bathing on these conditions. Following PRISMA guidelines and registration on the Open Science Framework (OSF), a systematic literature search was performed in major databases including PubMed, Embase, Web of Science, Scopus, Lilacs, IBECS, WPRIM, PsyINFO, and ProQuest in October 2024. Review Manager software was used to calculate mean differences (MD) between pre- and post-intervention outcomes, and the certainty of evidence was assessed using the GRADE. A total of 718 records were identified, of which 11 studies met the inclusion criteria. Forest bathing was associated with a reduction in heart rate (MD = −4.00; 95% CI = −6.90 to −1.09), tension-anxiety (MD = −0.79; 95% CI = −1.13 to −0.46), and depression despondency (MD = −0.68; 95% CI = −1.03 to −0.34), with confidence intervals that did not cross the effect line. However, heterogeneity among studies ranged from moderate to very high (I^2^ = 49%–87%), particularly for psychological outcomes. Although general effect tests demonstrated strong statistical significance for several psychological outcomes (*p* < 0.00001), the certainty of the evidence was classified as very low according to the GRADE, mainly due to the risk of bias, inconsistency, and imprecision. Forest bathing should be interpreted as a potential complementary practice for health promotion, and not as an individual preventive or therapeutic intervention for cardiovascular or mental disorders.

**Systematic review registration:**

https://doi.org/10.17605/OSF.IO/XETNK.

## 1 Introduction

In recent decades, the enormous technological, industrial, and economic advances achieved by modern society have led to profound transformations, driven primarily by the process of urbanization, which has brought mounting psychological pressures and circulatory problems to the majority of the world’s population (Kuddus et al., 2020; Fan, 2024). The United Nations [UN] (2022) estimated that more than half (50%) of the world’s population now lives in urban areas, and predicted that this percentage will increase to 70% by 2050. Most of this urban growth is occurring in cities of small and intermediate size, which often exacerbates inequalities and urban poverty (United Nations [UN], 2022).

In this context, practices that involve forests in the interface between health and the environment have been growing in popularity in recent years, reinforcing the Japanese concept of immersion in nature that arose in the 1980s, in particular, “Shinrin-Yoku” (forest bathing), which promotes immersion in nature as a form of promoting physical and mental wellbeing (Li, 2019). Shinrin-Yoku symbolizes the acceptance of the organic and psychological benefits derived from contact with forests, and is thus a practice that emphasizes the interdependence between human beings and the natural environment, extending its benefits to the conservation of biodiversity and the equilibrium of the planet (Kotera et al., 2022). More recently, Shinrin-Yoku was recognized as one of the most common practices of green tourism. Even so, there is also a need for this practice to be integrated with the preventive and curative care approaches that permeate human healthcare (Mammadova et al., 2021).

Chronic circulatory and mental diseases are associated increasingly with the urban environment. World Health Organization [WHO] (2025a,c) reported that cardiovascular disease (CVD) was the principal cause of global morbidity and mortality in 2024, with approximately 17.9 million deaths annually (Bikomeye et al., 2022). In 2019, mental disorders affected 970 million individuals, worldwide, and exceeded one billion in 2020, reflecting the impacts caused by the COVID-19 pandemic, with anxiety and depression being the most common disorders overall (World Health Organization [WHO], 2025b). Considering the ongoing and progressive increase in chronic, non-infectious diseases and the prevalence of mental disorders in modern society, Shinrin-Yoku offers a potential complementary strategy for the promotion of public health.

Interacting with natural environments can provide a range of health benefits, such as the reduction of stress, the balancing of blood pressure, and the regulation of the heart rate and the parasympathetic nervous system (Oh et al., 2017). It is important to note here that the isolation of human beings from natural environments has a direct impact on their health, the integral development of the individual, and the collective sustainability of natural resources, with serious implications for the preservation of biodiversity and the global ecological equilibrium. This scenario reinforces the approach to sociobiodiversity from a systemic approach, which considers all the possible interactions between the environment, human beings, and the place in which they live (Li, 2019).

Shinrin-Yoku practices and nature-based therapies represent an authentically holistic approach to the promotion of physical, mental, and social health (Kotera et al., 2022). Primary studies, systematic reviews, and meta-analyses have been employed to determine the preventive and therapeutic values of forest bathing, forest therapy, and forest medicine for a range of different conditions (Berman et al., 2008; Peen et al., 2010; van den Berg et al., 2010). Despite the lack of comparative scientific literature on the practice from the perspective of circulatory problems and mental disorders, there is a need to further synthesize the available evidence and, in particular, elucidate the efficacy of the procedure to guide decision-making on issues related to the preservation and certification of natural environments and the incorporation of healthcare technologies for the implementation of Shinrin-Yoku (van den Berg et al., 2010).

Given the ongoing growth of interest in experiences related to wellness and reconnection with nature, recreational activities based on the Shinrin-Yoku concept have become an important tourism sector (Chen et al., 2025), with increasing relevance for public health through their potential to enhance population-level access to nature-based interventions, support scalable low cost health promotion strategies. These activities are increasingly associated with human healthcare, which has consolidated the immersive aspect of forest bathing as a permanent, integrated therapeutic intervention (Guardini et al., 2023). Shinrin-Yoku is recommended not only for patients, but also for healthy individuals, due to its intrinsically preventive nature (Stier-Jarmer et al., 2021). Improving the quality of life and increasing wellbeing are particularly important objectives (Jimenez et al., 2021). The aim of this study was to systematically evaluate the short term cardiovascular and mental health responses, comparing the pre- and post-intervention periods with Shinrin-Yoku (forest bathing).

## 2 Methods

### 2.1 Study identification

The present study is based on a systematic review of the literature accompanied by a structured meta-analysis, which follows the parameters of the Preferred Reporting Items for Systematic Reviews and Meta-Analyses (PRISMA) protocol (Liberati et al., 2009), which is available on the Open Science Framework (OSF) platform, at https://doi.org/10.17605/OSF.IO/XETNK. The research question was based on the PICOS acronym, as follows: “What is the efficacy of forest bathing in the prevention and care of circulatory problems and mental disorders?” (Supplementary Chart 1).

The literature search strategy was compiled using the following Mesh descriptors and free terms: “Shinrin-Yoku,” “Forest bathing,” “Nature bathing,” “Forest therapy,” and “Nature Therapy.” These terms were applied through a structured approach, with the specific adjustments necessary for each of the different electronic databases consulted. The databases consulted here were: Medline via Pubmed, Embase, Web of Science, Scopus, Lilacs, the Spanish Language Health Sciences Bibliographic Index (IBECS), WPRIM (Western Pacific), PsyINFO, and ProQuest (see Supplementary Table 1). The search was conducted in October 2024.

### 2.2 Eligibility criteria

The inclusion criteria were based primarily on the type of study, with three types of study being considered here, that is: (i) randomized, controlled clinical trials, (ii) observational studies (cohort and case control), and (iii) quasi-experimental studies, which compared before and after scenarios, in terms of the outcomes of circulatory problems and mental disorders in diseased (healthcare) and non-diseased (prevention) individuals. The monitoring time was limited to a maximum of 24 h after the application of the intervention. No time restrictions or language limitations were applied in this review. Urban control groups were not analyzed in this study, as the priority was to assess the conditions of the groups exposed to forest bathing before and after exposure.

The exclusion criteria were as follows: case series, reviews, letters to the editor, qualitative studies with incomplete text; studies that did not provide a complete explanation of their statistical methods; studies that associated forest bathing with educational practices, therapeutic exercises or psychological support; studies that did not evaluate the efficacy of forest bathing, comparing the before and after conditions in the context of the prevention and care of circulatory problems or mental disorders, as well as studies that investigated only the components of the forest, the different types of forest separately, and virtual forest.

### 2.3 Selection of studies and data collection

The Mendeley version 1.18 references manager was used to organize the studies and remove all the duplicate references. The Rayyan QCRI^1^ platform was used to select the studies for the reading of the title and abstract by two independent researchers (A.M.A. and S.F.D.). All divergences were resolved independently by a third researcher (L.C.R.F.N.).

The data were extracted independently by two reviewers (A.M.A. and S.F.D) using a purpose-designed Microsoft Excel 2016 spreadsheet, with the discrepancies being resolved by consensus with a third researcher (L.C.R.F.N.). Whenever necessary, the authors of the selected studies were contacted to solicit additional information on the study, while experts in the fields of collective health, integrative practices, and the environment were also consulted. A standard set of data was extracted from each study and organized using a spreadsheet: (i) author, (ii) year of publication, (iii) country, (iv) objective, (v) population, (vi) type of study, (vii) intervention group, (ix) total sample (N), (x) study period, (xi) natural resources, (xii) control temperature, (xiii) dependent variables, (xiv) measurement tools, (xv) the activities developed during the study, and (xvi) financing. The studies excluded after the complete reading of the text are listed in Supplementary Table 2.

In the case of circulatory problems, the analysis of the outcomes considered the efficacy of the techniques applied in the study on (i) heart rate, (ii) pulse rate, and (iii) blood pressure (systolic and diastolic). For mental disorders, efficacy was evaluated based on (i) salivary cortisol (g.dL^1^), (ii) the Profile of Mood States [POMS: (1) Tension-anxiety; (2) Depression-despondency; (3) Anger hostility; (4) Fatigue; (5) Confusion; (6) Vigor], (iii) the Positive and Negative Affect Schedule (PANAS), (iv) the Restorative Outcome Scale (ROS), (v) the Subjective Vitality Scale (SVS), and (vi) the State Trait Anxiety Inventory A-State Scale (STAI-S). Salivary cortisol concentrations were reported using different units across the included studies. To ensure comparability and allow quantitative synthesis, cortisol values reported in nmol.L^–1^ were converted to g/dL^–1^ using the molar mass of cortisol (C_21_H_30_O_5_; 362.465). This unit conversion was performed solely for standardization purposes and did not alter the relative magnitude or direction of the observed effects.

### 2.4 Risk of bias and quality of the evidence

The methodological quality of the studies included in this assessment was evaluated using the Cochrane Collaboration, Risk of Bias (ROB) 2.0 tool and ROBINS I-V2 for non-randomized studies. The risk of bias was also assessed independently by two reviewers (A.M.A and S.F.D), and the divergences were resolved by consensus, together with a third reviewer (L.C.R.F.N.). The ROB 2.0 tool considers five domains of risk bias: (i) the randomization process; (ii) deviations from the intended interventions; (iii) the lack of data (results); (iv) measurement tools, and (v) the selection of the reported result. The risk of bias of each domain was classified as either low, some preoccupation or high (Sterne et al., 2019; Higgins et al., 2016).

The ROBINS I-V2 tool is composed of seven domains of bias risk: (i) bias due to confusion; (ii) bias in the selection of the participants; (iii) bias in the classification of the intervention; (iv) bias derived from deviations from the intended intervention; (v) bias in the measurement of the outcome; (vi) bias derived from incomplete data on the outcome, and (vii) bias in the selection of the result reported. The risk of bias for each domain was scored as (a) low, (b) moderate, (c) serious or (d) critical (Cochrane Database of Systematic Reviews, 2024).

The online GRADEpro software^2^ was used to evaluate the quality of the evidence. The Grading of Recommendations Assessment, Development and Evaluation (GRADE) provides a critical approach for the evaluation of the evidence presented for each outcome, considering four different levels, which represent the level of confidence in the estimates of the effects reported – (a) very low, (b) low, (c) moderate, and (d) high (Brasil, 2014).

### 2.5 Statistical analysis

The Review Manager^®^ software (version 5.1.4) was used to conduct the meta-analyses, explicitly accounting for the paired (pre–post) nature of the outcome measurements, since all interventions were applied to the same individuals. For each continuous outcome, effect sizes were calculated based on the mean change score (post-intervention minus pre-intervention) within each study. When directly reported, the mean difference and standard deviation of the change were extracted. When change-score standard deviations were not available, they were estimated from pre- and post-intervention standard deviations assuming a conservative within-subject correlation, in accordance with the Cochrane Handbook recommendations. Mean Difference (MD) with 95% confidence intervals (CI) was used, given that outcomes were measured on comparable scales across studies. A random-effects model was applied due to expected clinical and methodological heterogeneity related to population characteristics, intervention protocols, and outcome assessment tools.

For all meta-analyses, effect sizes were calculated exclusively using the Mean Difference (MD) with 95% confidence intervals (CI). This approach was adopted because pooled outcomes were derived only from studies that used identical measurement instruments, with the same scale ranges, units, and clinical interpretation, ensuring direct comparability across studies. Cardiovascular outcomes (e.g., heart rate and blood pressure) and psychological outcomes assessed with the same questionnaire were synthesized separately for each instrument.

Psychological measures assessed using different validated tools with distinct scale properties (POMS, PANAS, ROS, STAI-S and SVS) were not combined into a single pooled estimate, in order to minimize the risk of measurement-related bias and loss of clinical interpretability. When fewer than two studies used the same psychological instrument (BDI, EQ5D, MAAS-C, CNS, and DS method), results were summarized narratively rather than meta-analytically.

Statistical significance of the intervention effects was determined based on the overall test of effect provided by the Z statistic in RevMan, with *p* < 0.05 indicating a statistically significant pooled mean difference. Confidence intervals not crossing zero were interpreted as evidence of a statistically significant effect, regardless of heterogeneity levels. Statistical heterogeneity was assessed separately using Cochran’s Q test, with the corresponding *p*-value referring exclusively to between-study inconsistency rather than intervention efficacy. Heterogeneity was quantified using the I^2^ statistic and classified as low (I^2^ < 25%), moderate (25%–50%), or high (>50%) (Higgins and Thompson, 2002).

## 3 Results

### 3.1 Characteristics of the studies

**FIGURE 1:**
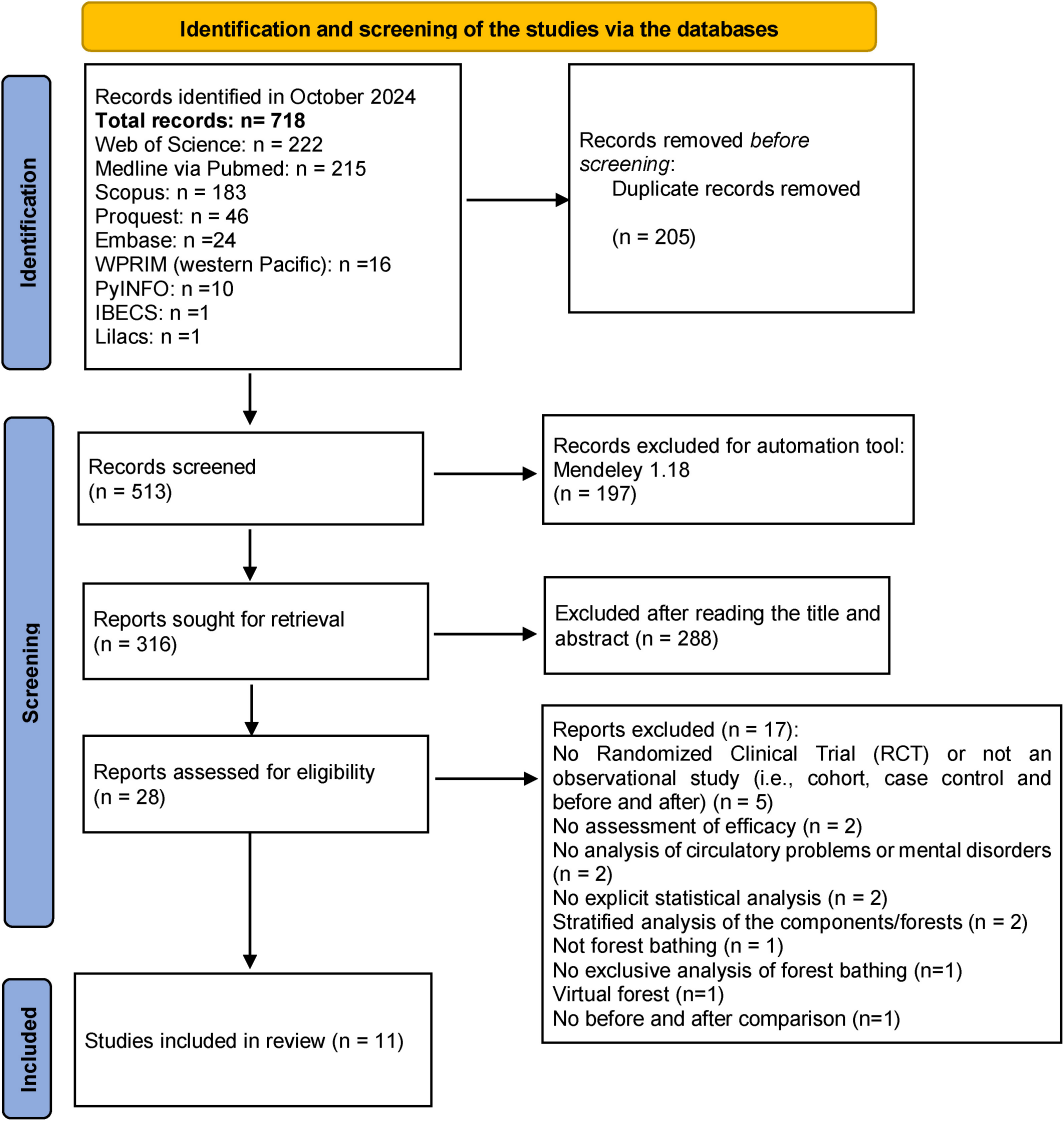
Flowchart of the selection of the studies for analysis the present study, adapted from the PRISMA statement (2020). Source: elaborated by the authors.

The literature search identified a total of 718 publications, of which, 11 studies were selected for analysis (Figure 1). Ten of these 11 studies were classified as quasi-experimental (with before and after comparisons).

The before and after conditions of the exposure to forest bathing were compared primarily in the quasi-experimental studies (90.1% of the total). Most of the scientific evidence (63.6%) identified in this systematic review had been compiled over the past 6 years, and 45.5% of the studies were developed in a single day. A majority (63.6%) of the published studies were conducted in either Poland or Japan, while no studies were reported from Africa, Oceania or Central or South America (Table 1).

**TABLE 1:** Characterization of studies included in the systematic review (*n* = 11).

References	Country	Type of study	Study period	Population	Sample (*N*)	Intervention group (forest bath)	Temperature	Activities developed
Quan et al., 2024	China	Quasi-experimental study	July 6–8th 2019	Men and women (non-diseased) with a mean age of 37.1 years (NR^1^)	*N* = 12	Forest: lotus flowers, thermal water in the forest	14 °C–18.5 °C	Walk (NR^6^)
Chun et al., 2023	South Korea	Quasi-experimental study	August 15th 2020	Men and women (non-diseased) with a mean age of 32.7 years (NR^1^)	*N* = 99	Forest: trees with birds	Not recorded	2-h walk
Kil et al., 2023	United States	Quasi-experimental study	July 2019	Boys and girl (diseased), 9–14 years old (NR^1,2^)	*N* = 12	Forest: municipal forest park with natural soil-based trails and diverse wildlife and natural elements	Not recorded	Walks of approximately 1 h
Bielinis et al., 2021	Finland	Quasi-experimental study	January 29th 2019	Male and female^3^ (non-diseased) with a mean age of 22.5 (±4.67) years^3^	*N* = 22	Forest: environment in which the ground and trees were covered with snow	Forest: −7.23 °C	5-min walk to the site, with 15 min of immersion (sitting/standing/moving)
Janeczko et al., 2020	Poland	Quasi-experimental study	29 November 2019	Male and female^3^ (non-diseased) with a mean age of 24 years^3^	*N* = 75 (forest: *n* = 30; urban area: *n* = 45)	Forest: Kabaty urban forest and Sobieski with coniferous and deciduous trees	Forest: 8 °C urban area: 9.2 °C	30-min walks
Bielinis et al., 2019	Poland	Quasi-experimental study	July 27th to November 29th 2018	Male and female^3^ (diseased) with a mean age of 42.44 (±13.23) years^3^	*N* = 50	Forest: suburban forest covered principally with 65–180 year-old Scots pines (*Pinus sylvestris* L.), with some 95–105 year-old Norwegian spruce (*Picea abies* (L.) H. Karst.) and 95–110 year-old pedunculate oak (*Quercus robur* L.), and some 15 year-old beech (*Fagus sylvatica* L.)	Forest: 20 °C–25 °C	Walks of 1 h and 45 min
Kobayashi et al., 2019	Japan	RCT switch^4^	Not recorded	Male^3^ (non-diseased) with a mean age of 22.4 (±1.8) years^3^	*N* = 74	Forest: seven Japanese forests^5^	Not recorded	15-min walk
Bielinis et al., 2018	Poland	Quasi-experimental study	March 2nd 2017	Male and female^3^ (non-diseased) with a mean age of 21.45 (±0.18) years	*N* = 62	Forest: urban, deciduous, broadleaf, including *F. sylvatica*, *Q. robur*, and black alder (*Alnus glutinosa* (L.) Gaertn.).	Forest: 4 °C urban area: 9.2 °C	15-min walk and 15 min visit (standing)
Ochiai et al., 2015	Japan	Quasi-experimental study	August 30th 2014	Women (non-diseased) with a mean age of 62.2 (±9.4) years	*N* = 17	Forest: Akasawa, Agematsu natural recreation area	Forest: 21.5 °C	Multiple timed activities over a 4:41 h period (walks/rests/sitting and lying down)
Lee et al., 2011	Japan	Quasi-experimental study	September 12–14th 2016	Men and women (diseased/non-diseased) with a mean age of 21.2 (±0.9) years	*N* = 12	Forest: broadleaf deciduous trees in Tsurui village	Not recorded	15-min observation (sitting)
Morita et al., 2007	Japan	Quasi-experimental study	November and December 2006	Men and women (non-diseased) with a mean age of 56.2 (±10.6) years	*N* = 498	Forest: Japanese forest	Not recorded	1-h walk

^1^Not recorded (NR) = mean not provided.

^2^Not recorded (NR) = standard deviation not provided.

^3^Undergraduate students.

^4^RCT switch: randomized clinical trial with exchange of participants with equal N for both groups.

^5^Forests not detailed.

^6^Not recorded (NR) = duration not provided. Source: elaborated by the authors.

The principal portion of the population analyzed in the primary studies were male and female non-diseased (63.6%), with mean ages ranging from 21.2 (±0.9) years to 56.2 (±10.6) years. The forests in which the activities were developed present multiple natural resources with a number of different types of vegetation, although six of the 11 studies (63.6%) were conducted in Asian forests, and two were in urban/suburban forests, while in one case, the ground and trees were covered in snow. The reported temperatures ranged from −7.23 °C to 25 °C, although five of the 11 studies (45.5%) did not provide information on the ambient temperature, while walking was an activity developed in almost all (90.1%) of the studies (Table 1).

The objectives of the study included outcomes related to both circulatory problems and mental disorders in a majority (63.6%) of the studies analyzed. Eight (72.7%) of the studies received financial support, which was provided by the university in half of the cases (Supplementary Table 3).

### 3.2 Risk of bias and the quality of the evidence

**FIGURE 2:**
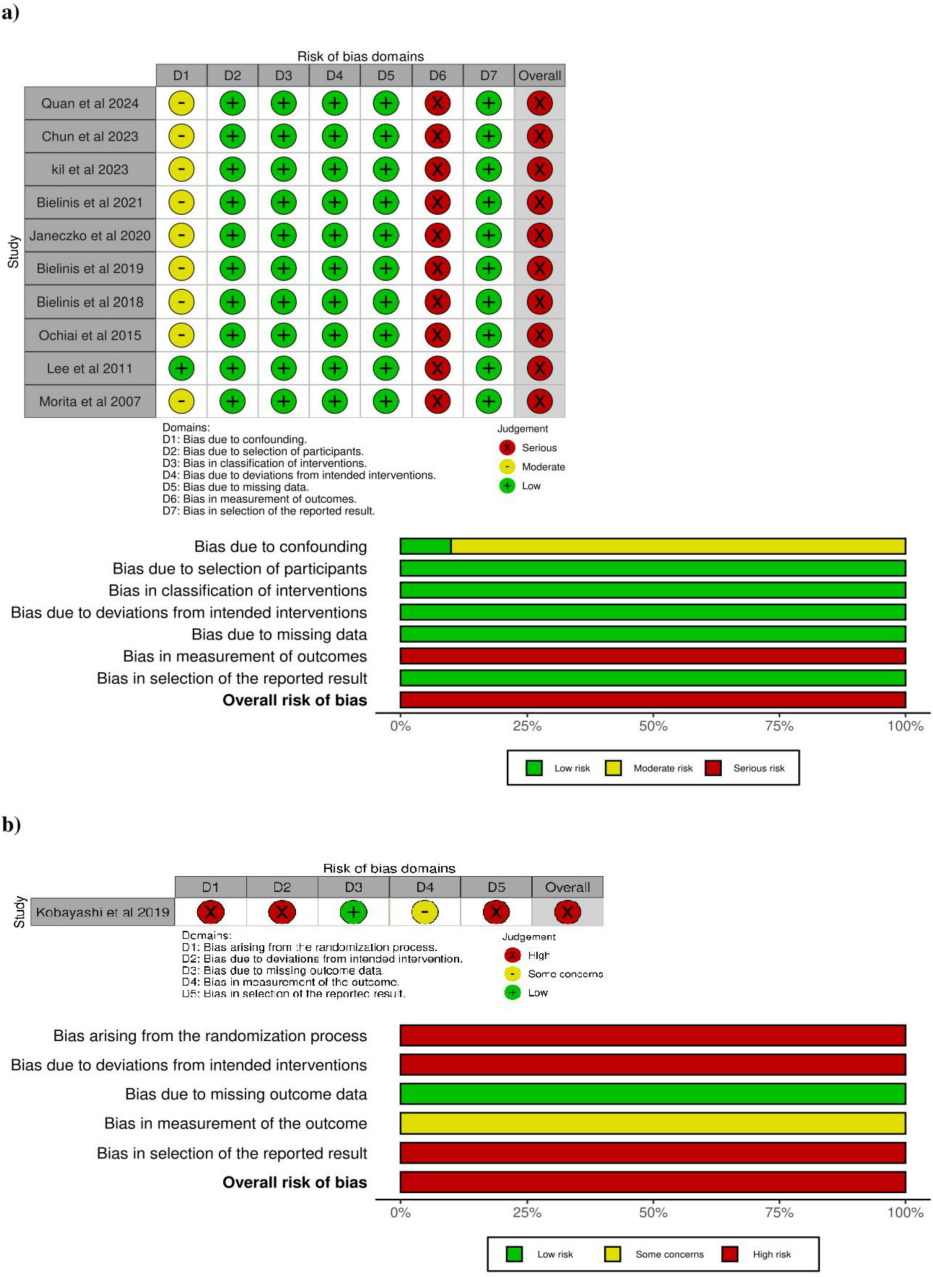
Risk of bias in the studies assessed in the systematic review presented here, with the meta-analysis based on the **(a)** ROBINS I-V2, and **(b)** ROB 2.0 tools. Source: elaborated by the authors.

All the non-randomized studies were assessed as having a serious risk of bias, while the randomized study was found to have a high risk of bias (Figure 2). The critical aspects of the ROBINS I-V2 analysis indicate serious risks in the bias domain related to confusion (Quan et al., 2024; Chun et al., 2023; Kil et al., 2023; Bielinis et al., 2019, 2021; Janeczko et al., 2020; Morita et al., 2007, given the lack of control for the bias of the walk, as well as being applied during the winter (Bielinis et al., 2018, 2021). There was also a high level of heterogeneity in the age range (Kil et al., 2023; Bielinis et al., 2019; Ochiai et al., 2015; Morita et al., 2007), as well as in studies that focused only on individuals of the female sex (Ochiai et al., 2015). The bias domain related to the incomplete outcome data, which presented a serious risk of bias, given the lack of a blind approach for the participants, research team, and other investigators involved in the study (Quan et al., 2024; Chun et al., 2023; Kil et al., 2023; Bielinis et al., 2018, 2021; Janeczko et al., 2020; Morita et al., 2007).

In the case of the critical aspects of the ROB 2.0 analysis, as in the case of the study of Kobayashi et al. (2019), in the domain of the randomization process, the confidentiality of the allocation procedure was not clear. In the domain of the deviations from the intended interventions, the procedure was not kept blind from the participants, research team, and other investigators of the study. In the domain of data quantification, the analysis of only individuals of the male sex generated a high level of heterogeneity. In the domain of the selection of the results reported, the protocol of clinical records was not cited.

The quality of the evidence was considered to be very low for all the outcomes in the GRADE analysis. In particular, there was a reduction of one point for the factors (i) risk of bias, due to the lack of masking (blind trials), (ii) inconsistencies due to the heterogeneity of the study population, and (iii) publication bias derived from the confusion factors that may have reduced the effects demonstrated at the low winter temperature and the walk that was not controlled, as well as the types of observation, i.e., sitting, lying down, and standing (Supplementary Table 4).

### 3.3 Efficacy

#### 3.3.1 Physiological outcomes related to circulatory problems

**FIGURE 3:**
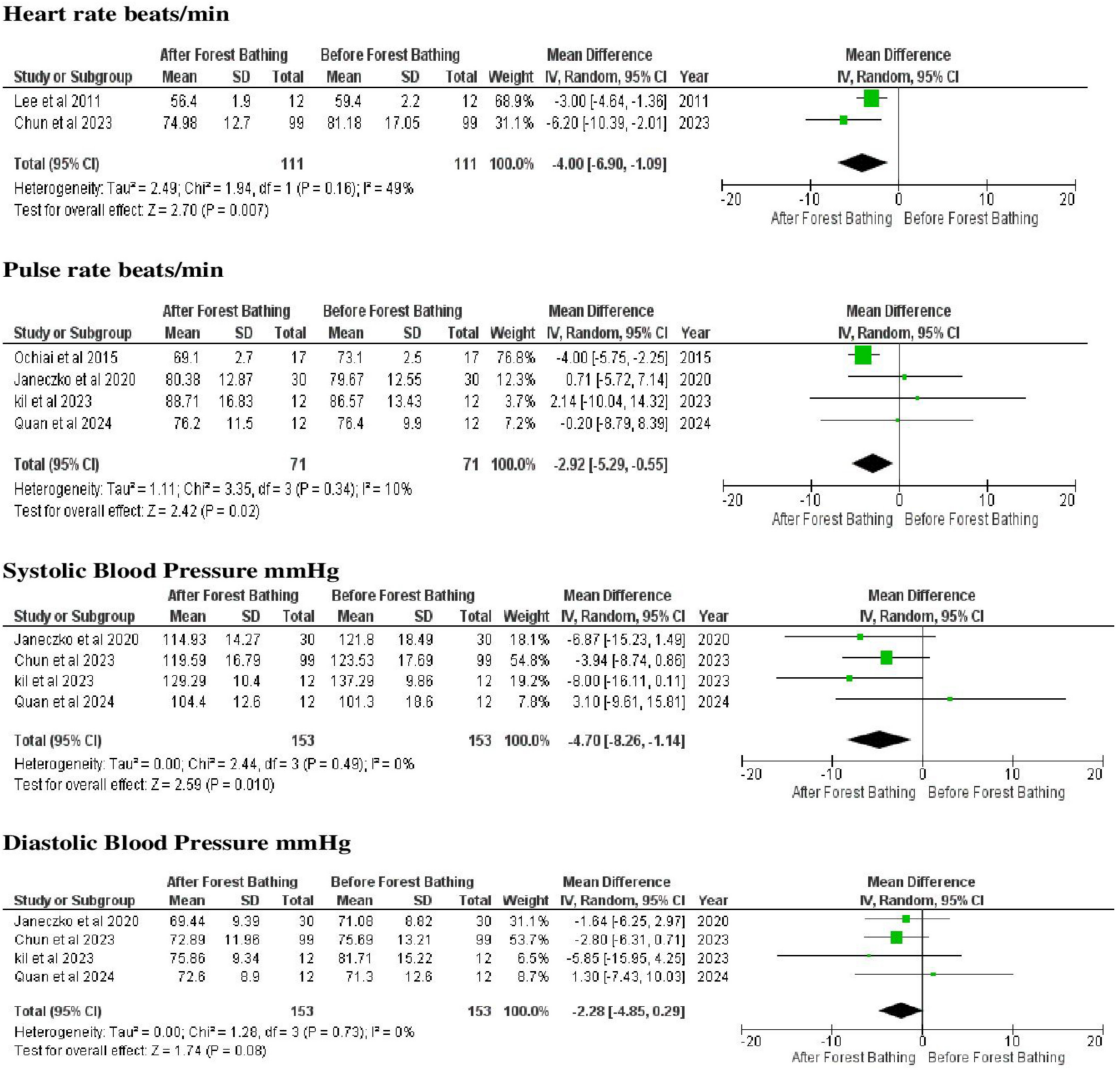
Meta-analyses of the heart rate and blood pressure (systolic and diastolic) and pulse rate data related to the circulatory problems, as identified in the studies reviewed here. Source: elaborated by the authors.

Physiological outcomes related to circulatory problems were assessed based on heart rate, pulse rate, and blood pressure (systolic and diastolic), measured using mechanical or digital instruments (Supplementary Table 3). Forest bathing was associated with reductions in heart rate (MD = −4.00; 95% CI = −6.90 to −1.09), pulse rate (MD = −2.92; 95% CI = −5.29 to −0.55), and systolic blood pressure (MD = −2.92; 95% CI = −5.29 to −0.55), as indicated by confidence intervals that do not cross the effect line. Heterogeneity among studies for these outcomes ranged from low to moderate (I^2^ = 10%–49%). The reported *p*-values (0.14–0.34) refer to Cochran’s Q test for heterogeneity, indicating a lack of statistical significance (Figure 3). However, no significant difference was observed in diastolic blood pressure (DM: −2.28; 95% CI = −4.85 to 0.29), since the confidence interval crossed zero, I^2^ = 0% and *p* = 0.73 (Figure 3).

#### 3.3.2 Psychological outcomes related to mental disorders

The meta-analysis assessed psychological outcomes including tension–anxiety, depression–despondency, anger–hostility, fatigue, confusion, vigor, restorative effect of the environment, vitality, and positive and negative emotional affect. These outcomes were measured using validated instruments such as (Supplementary Table 2). Depression was additionally assessed using salivary cortisol, and anxiety using the STAI (Supplementary Table 3).

**FIGURE 4:**
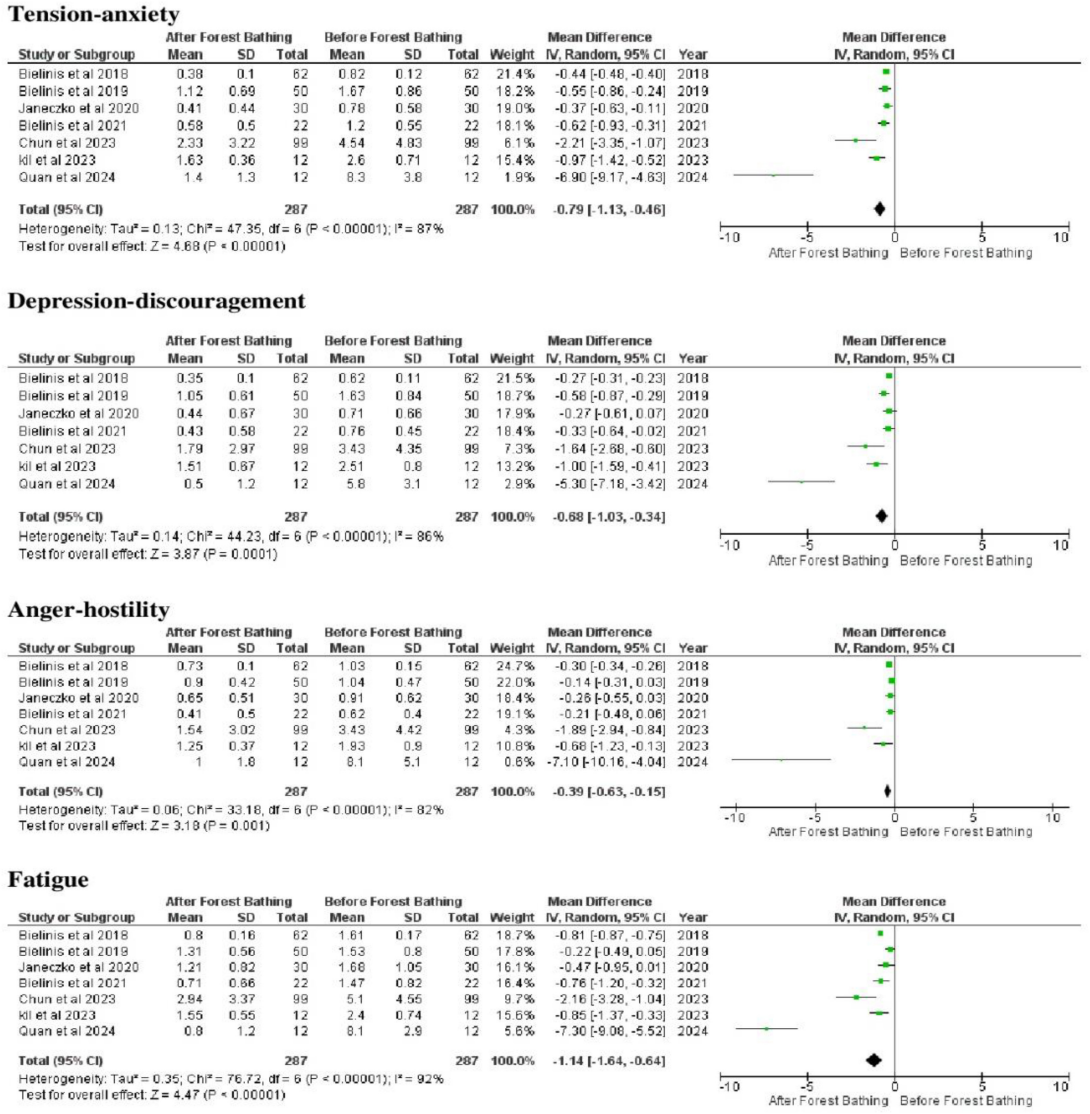
Meta-analyses of the tension-anxiety, depression-despondency, anger-hostility, and fatigue related to the mental disorders. Source: elaborated by the authors.

Forest bathing was associated with reductions in tension–anxiety (MD = −0.79; 95% CI = −1.13 to −0.46), depression–despondency (MD = −0.68; 95% CI = −1.03 to −0.34), anger–hostility (MD = −0.39; 95% CI = −0.63 to −0.15), fatigue (MD = −1.14; 95% CI = −1.64 to −0.64), confusion (MD = −0.68; 95% CI = −0.99 to −0.37), and negative emotional affect (MD = −0.08; 95% CI = −0.14 to −0.01), as confidence intervals excluded the effect (Figure 4 and Supplementary Figures 1, 3). The overall tests of effect for these psychological outcomes showed strong statistical evidence of an intervention effect (*p* < 0.00001). Heterogeneity across studies ranged from low to very high (I^2^ = 10%–92%), and *p* = 0.00001–0.33, reflecting variability in study results.

Conversely, vigor (MD = 0.62; 95% CI = 0.29–0.94) and restorative effect of the environment (MD = 1.09; 95% CI = 0.69–1.48) increased following forest bathing. Heterogeneity was high for vigor (I^2^ = 84%; *p* < 0.00001) and moderate for restorative effect (I^2^ = 48%; *p* = 0.15) (Supplementary Figures 2, 5). Salivary cortisol showed no clinically meaningful change (MD = 0.09; 95% CI = 0.03–0.15), despite very high heterogeneity (I^2^ = 99%; *p* < 0.00001) (Supplementary Figure 7).

No clear evidence of change was observed for vitality (MD = 0.61; 95% CI = −0.36 to 1.57), positive emotional affect (MD = 0.24; 95% CI = −0.12 to 0.59), or anxiety assessed by STAI (MD = −6.15; 95% CI = −15.71 to 3.40), as confidence intervals crossed the line of effect. The high I^2^ values (79%–98%) and *p* = 0.00001–0.009 indicate high variability in studies (Supplementary Figures 4, 8).

Additional psychological instruments analyzed individually showed improvements following forest bathing: the Beck Depression Inventory (BDI) demonstrated reduced depressive symptoms (*p* = 0.01), the EuroQol-5 Dimension (EQ-5D) indicated improved quality of life (*p* = 0.01), the Mindfulness Attention Awareness Scale-Children (MAAS-C) showed increased mindfulness (*p* = 0.007), and the Connectedness to Nature Scale (CNS) revealed enhanced nature connectedness (*p* < 0.001). The Differential Semantic (DS) method indicated improvements in perceived comfort, relaxation, and revitalization (all *p* < 0.01) (Supplementary Table 3).

### 3.4 Analysis of sensitivity

Analyses of sensitivity were conducted to explore the potential outcomes of the subgroup that analyzed diseased and non-diseased individuals. The exclusion of studies of diseased and non-diseased populations did not produce a significant effect different Mean Difference (MD) in the majority of cases, i.e., heart rate, pulse rate, and diastolic blood pressure for outcomes related to circulatory problems, and tension-anxiety, depression-despondency, confusion, vigor, the restoring effect of each environment, vitality, and the positive and negative emotional affects for outcomes related to mental disorders (Supplementary Figures 3–6, 8–13, 16–23). However, showed evidence of effect in the non-diseased subgroup for anger-hostility (MD: −0.52; 95% CI = −0.91 to −0.13; I^2^ = 86%; *p* < 0.0001) and fatigue (MD: −1.69; 95% CI = −2.50 to −0.88; I^2^ = 93%; *p* < 0.00001) (Supplementary Figures 14, 15).

## 4 Discussion

Despite the growing interest in healthcare practices involving caregiving processes related to forest environments, such approaches still play a negligible role in healthcare systems worldwide (Krala-Szkaradowska et al., 2024). In this context, the findings of this systematic review and meta-analysis suggest that forest bathing may be associated with favorable short-term physiological and psychological effects, rather than demonstrating a definitive role in the prevention or management of circulatory and mental disorders. These results indicate a potential complementary contribution of forest bathing within integrative and health-promoting approaches. Nevertheless, its broader implementation faces important challenges, including political and bureaucratic barriers and limited dissemination of scientific evidence among decision-makers, which constrain the incorporation of emerging health practices into formal clinical and public health protocols (Vermeesch et al., 2024).

The results of the present review showed clearly that there was a significant reduction in heart rate, pulse rate, and systolic blood pressure following the forest bathing intervention, which reinforces the relevance of this practice as an integrative management tool in cardiovascular healthcare. These findings are also consistent with those of previous studies (Garibay-Chávez et al., 2024; Wen et al., 2023), which have documented the relaxing effects of forest bathing on the autonomous nervous system, promoting a predominance of the parasympathetic tonus and reducing sympathetic activity, processes that are linked closely with the control of hemodynamic parameters, and may contribute to transient decreases in cardiac workload, vascular resistance, and perceived physiological arousal. Although heterogeneity for some cardiovascular outcomes was low to moderate (I^2^ = 10%–49%), the observed effects cannot be directly extrapolated to clinical prevention or management of hypertension, particularly given the absence of long-term follow-up and standardized intervention protocols.

No significant changes were observed in diastolic blood pressure, suggesting that the potential physiological effects of forest bathing may be limited to specific cardiovascular parameters (Simpattanawong et al., 2024). This inconsistency may be explained by individual characteristics such as age, baseline health status, or intervention intensity, as well as by the walking component inherent to most forest bathing protocols (Peterfalvi et al., 2021; Garibay-Chávez et al., 2024). Walking is a simple, accessible, and low cost practice, which is known to have a number of benefits for the physical health of the individual (Simpattanawong et al., 2024). In particular, Iwane et al. (2000) found that walking at least 10,000 steps per day may help to reduce blood pressure and the activity of the sympathetic nervous system in individuals with hypertension. Even so, there is still no consensus on the exact effects of forest bathing itself, independently of the walking component.

The psychological outcomes of forest bathing revealed significant benefits in terms of the reduction of the indicators (tension-anxiety, depression-despondency, anger-hostility, fatigue, confusion, and negative emotional affects) related to mental disorders. Queirolo et al. (2024) and Brahmaiah et al. (2024) found that the natural environment had a restorative impact on mental wellbeing, with improvements in emotional control and a reduction in the symptoms of stress. However, these findings were characterized high heterogeneity, reflecting marked variability across study populations, intervention designs, exposure durations, and environmental contexts. Such heterogeneity substantially limits the certainty and generalizability of these associations (Table 1).

Increases in vigor and perceived restorative effects of the environment were also observed, suggesting that forest bathing may contribute to subjective feelings of revitalization in addition to relaxation (Siah et al., 2023). In contrast, salivary cortisol levels showed negligible changes, which may indicate either an adaptive physiological response requiring longer exposure or methodological inconsistencies across studies (Antonelli et al., 2019). The data also revealed a high level of heterogeneity observed for cortisol outcomes further reinforces the uncertainty surrounding biological stress markers in this context, potentially influenced by sex differences and baseline stress profiles (Kobayashi et al., 2019; Ochiai et al., 2015).

Neither vitality, positive emotional affects nor anxiety varied significantly following the intervention. Subirana-Malaret et al. (2023) and Langer et al. (2023) inferred that factors such as the time of exposure, the demographic traits of the participants or variation in the procedures adopted in the different studies may differentiate the results in a small number of cases. The high level of heterogeneity observed in these outcomes indicates that future studies should aim to achieve a greater uniformity in the age classes of the subjects, in order to ensure more robust comparisons for a better assessment of the possible modulators of the efficacy of forest bathing.

Improvements in depression scores measured by the BDI and in quality of life assessed by the EQ-5D indicate potential psychosocial benefits, particularly among vulnerable populations (Chun et al., 2023). Similarly, gains in mindfulness and connectedness to nature observed through the MAAS-C and CNS scales are consistent with prior literature linking natural exposure to enhanced cognitive and emotional engagement (Antonelli et al., 2021). The DS method must be applied more widely in experimental studies, these effects have yet to be confirmed in an analysis of multiple studies (Farrow and Washburn, 2019).

Nonetheless, these findings remain exploratory, as several psychological tools were applied in single studies only, limiting their contribution to pooled evidence. Importantly, disparities in access to green spaces may constrain the applicability of such interventions among socioeconomically disadvantaged or urban populations, raising equity considerations that warrant explicit attention in future research and policy planning (Chun et al., 2023).

Overall, it is important to note that the analyses of sensitivity indicated a lack of any effect on anger-hostility or fatigue in the diseased subgroup, and on systolic blood pressure in the non-diseased subgroup. These results underscore the need for subgroup-specific analyses and caution against broad generalizations regarding the effects of forest bathing across populations (Li, 2022).

### 4.1 Limitations

Several limitations were identified in this systematic review, including reduced quantification in the primary experimental studies, small sample sizes, and variation in forest bathing exposure protocols (both in terms of intervention intensity and outcome measurement). Inconsistencies were also observed between pre- and post-intervention variables such as walking schedules and seasonal factors, which introduced bias, particularly in studies conducted during winter. A significant methodological limitation is that most included studies adopted uncontrolled pre-post designs, without comparison to control groups in urban or alternative settings. This limits causal inference and requires results to be interpreted as short-term associations rather than definitive intervention effects. In many cases, moreover, the samples were not very representative, due to the predominance of only one sex, for example, and the wide variation in age ranges, which compromised the extrapolation of results to the general population. The lack of blinded sampling of participants, the research team, and other investigators, was a recurring problem in all the studies analyzed, increasing the risk of bias in data collection and interpretation. There was also a lack of clarity regarding the confidentiality of allocation, while previous records of the clinical protocol compromised methodological quality in essential areas, such as deviations from planned interventions and the measurement of parameters in the randomized clinical trial. The GRADE assessment indicated that the certainty of evidence for all outcomes was classified as very low, mainly due to the risk of bias, inconsistency, and imprecision, which substantially limits the strength of inferences and the practical applicability of the conclusions.

### 4.2 Implications for research and clinical practice

The implications for research derived from the present analysis include an emphasis on the need to delineate standardized parameters for Shinrin-Yoku interventions, as well as the potential need to explore subjacent biological mechanisms, such as the roles of the phytoncide and the environmental microbiota (Li et al., 2016). The increasing acceptance of Shinrin-Yoku by the medical community has led to the implementation of healthcare programs that prescribe nature, in which the physicians recommend walks in areas of natural vegetation as part of the treatment of the patient.

For clinical practice, interventions based on forest bathing can be incorporated as complementary measures for the treatment of hypertension and chronic stress. The psychological effects of Shinrin-Yoku can also help reduce the symptoms of anxiety and depression, highlighting the importance of non-pharmacological therapeutic strategies for more vulnerable populations (Antonelli et al., 2021; Kotera et al., 2022). Shinrin-Yoku has been integrated increasingly with complementary therapeutic approaches for the management of psychiatric disorders and chronic diseases. Interventions based on nature therapy have shown promising results in the treatment of depression, anxiety disorders, burnout syndrome, and arterial hypertension. In addition, forest bathing can be adapted in a clinical context to different cultural and geographic environments, amplifying the reach and applicability of the care system.

## 5 Conclusion

The results of the systematic review and meta-analysis reinforce the fundamental principles of Shinrin-Yoku in the short term, with potential to improve clinical health practices and for the prevention and treatment of cardiovascular problems and mental disorders. However, the perception of forest bathing as a predominantly recreational activity persists, indicating the need for greater involvement of health professionals and environmental management to broaden the application of this procedure and expand its inclusion in health networks through an intersectoral approach. From a public health perspective, Shinrin-Yoku may also be understood as a preventive and wellness-oriented strategy, particularly within integrative and health-promoting models that emphasize lifestyle modification and environmental determinants of health.

This study also confirmed that forest environments stimulate parasympathetic activity and reduce sympathetic activity, in addition to promoting emotional benefits. However, the effects of these experiences vary among individuals due to their personal characteristics and the particularities of each environment. Despite the promising results of this study, the relatively short duration of the investigation, along with the small number of participants in the primary studies, reinforcing the need for studies with larger samples and longer exposure periods. Nonetheless, recognizing forest bathing as a multidisciplinary approach will require collaboration between areas as diverse as forestry, medicine, biology, psychology, public policy management, and environmental education.

Science has provided promising evidence of the benefits offered by Shinrin-Yoku as a potentially restorative and health-promoting practice associated with acute responses. Given the growing demand for non-pharmacological strategies for managing stress and mental health disorders, valuing practices based on the connection between humans and nature may be essential for the development of more sustainable and integrative health policies.

## Data Availability

The datasets presented in this study can be found in online repositories. The names of the repository/repositories and accession number(s) can be found in the article/Supplementary material.

## Footnotes

1
https://rayyan.qcri.org

2
https://gradepro.org/
