# Supplementary material for: Short-term cardiovascular and mental health responses to Shinrin-Yoku (forest bathing): a systematic review and meta-analysis
Source: Front Psychol. 2026 Feb 11;17:1707829. doi: 10.3389/fpsyg.2026.1707829 (PMC12932220; doi:10.3389/fpsyg.2026.1707829)
Supplement: Supplementary file 1 [file Table_1.DOCX]

**Supplementary Material**

**Chart 1.** PICOS framework for the systematic review and meta-analysis.

| **PICOS** | |
| --- | --- |
| P | People of any age, with and without circulatory problems and mental disorders |
| I | After forest bathing |
| C | Before forest bathing |
| O | To compare the efficacy compared before and after of forest bathing in the prevention and care of circulatory disorders and mental disorders |
| S | Randomized Clinical Trials (RCT) and Observational studies (cohort, case control and before and after) |

Source: Authors' elaboration

**Table 1.** Search strategy for the systematic review and meta-analysis.

| Bases de Dados | Estratégia | Total |
| --- | --- | --- |
| Medline via Pubmed | (Shinrin-Yoku) OR (forest bathing) OR (nature bathing) | 1.851 |
|  | AND |  |
|  | (forest therapy) OR (Relaxation Therapy) OR (Therapeutic Relaxation) OR (Relaxation, Therapeutic) OR (Therapy, Relaxation) OR (Relaxation Techniques) OR (Relaxation Technique) OR (Technique, Relaxation) OR (Techniques, Relaxation) OR (Relaxation Technics) OR (Relaxation Technic) OR (Technic, Relaxation) OR (Nature Therapy) OR (Nature Therapies) OR (Therapy, Nature) OR (Ecotherapy) OR (Ecotherapies) OR (Naturopathy) OR (Medicine, Naturopathic) OR (Naturopathic Medicine) | 184.542 |
|  | Total | 215 |
| Embase | ('forest bathing'/exp OR 'forest bathing' OR 'shinrin yoku' OR 'shinrin`yoku' OR 'nature bathing') | 147 |
|  | AND |  |
|  | ('forest therapy'/exp OR 'relaxation training'/exp OR 'mind-body and relaxation techniques' OR 'relaxation method' OR 'relaxation technics' OR 'relaxation techniques' OR 'relaxation therapy' OR 'relaxation training' OR 'training, relaxation') | 14.944 |
|  | Total | 24 |
| Web of Science | (Shinrin-Yoku) OR (forest bathing) OR (nature bathing) | 1.182 |
|  | AND |  |
|  | (forest therapy) OR (Relaxation Therapy) OR (Therapeutic Relaxation) OR (Relaxation, Therapeutic) OR (Therapy, Relaxation) OR (Relaxation Techniques) OR (Relaxation Technique) OR (Technique, Relaxation) OR (Techniques, Relaxation) OR (Relaxation Technics) OR (Relaxation Technic) OR (Technic, Relaxation) OR (Nature Therapy) OR (Nature Therapies) OR (Therapy, Nature) OR (Ecotherapy) OR (Ecotherapies) OR (Naturopathy) OR (Medicine, Naturopathic) OR (Naturopathic Medicine) | 203.493 |
|  | Total | 222 |
| Scopus | (Shinrin-Yoku) OR (forest bathing) OR (nature bathing) | 1.003 |
|  | AND |  |
|  | (forest therapy) OR (Relaxation Therapy) OR (Therapeutic Relaxation) OR (Relaxation, Therapeutic) OR (Therapy, Relaxation) OR (Relaxation Techniques) OR (Relaxation Technique) OR (Technique, Relaxation) OR (Techniques, Relaxation) OR (Relaxation Technics) OR (Relaxation Technic) OR (Technic, Relaxation) OR (Nature Therapy) OR (Nature Therapies) OR (Therapy, Nature) OR (Ecotherapy) OR (Ecotherapies) OR (Naturopathy) OR (Medicine, Naturopathic) OR (Naturopathic Medicine) | 189.650 |
|  | Total | 183 |
| PyINFO | “Shinrin-Yoku” OR “forest bathing” OR “nature bathing” | 21 |
|  | AND |  |
|  | “forest therapy” OR “Relaxation Therapy” OR “Therapeutic Relaxation” OR “Relaxation, Therapeutic” OR “Therapy, Relaxation” OR “Relaxation Techniques” OR “Relaxation Technique” OR “Technique, Relaxation” OR “Techniques, Relaxation” OR “Relaxation Technics” OR “Relaxation Technic” OR “Technic, Relaxation” OR “Nature Therapy” OR “Nature Therapies” OR “Therapy, Nature” OR “Ecotherapy” OR “Ecotherapies” OR “Naturopathy” OR “Medicine, Naturopathic” OR “Naturopathic Medicine” | 6.951 |
|  | Total | 10 |
| WPRIM (Pacífico Ocidental) | (Shinrin-Yoku) OR (forest bathing) OR (nature bathing) OR (banho de floresta) OR (baño de bosque) OR (baignade en forêt) | 35 |
|  | AND |  |
|  | (forest therapy) OR (Relaxation Therapy) OR (Therapeutic Relaxation) OR (Relaxation, Therapeutic) OR (Therapy, Relaxation) OR (Relaxation Techniques) OR (Relaxation Technique) OR (Technique, Relaxation) OR (Techniques, Relaxation) OR (Relaxation Technics) OR (Relaxation Technic) OR (Technic, Relaxation) OR (Nature Therapy) OR (Nature Therapies) OR (Therapy, Nature) OR (Ecotherapy) OR (Ecotherapies) OR (Naturopathy) OR (Medicine, Naturopathic) OR (Naturopathic Medicine) OR (terapiadefloresta) OR (Terapia de Relaxamento) OR (Terapia por Relajación) OR (ecoterapia) OR (naturoterapia) OR (relajación terapéutica) OR (técnicas de relajación) OR (Thérapie par la relaxation) OR (Ecoterapia) OR (Relaxamento Terapêutico) OR (Terapia Apoiada pela Natureza) OR (Terapia Assitida pela Natureza) OR (Terapia da Natureza) OR (Terapia por Relaxamento) OR (Terapia por meio da Natureza) OR (Técnicas de Relaxamento) | 1.968 |
|  | Total | 16 |
| IBECS | (Shinrin-Yoku) OR (forest bathing) OR (nature bathing) OR (banho de floresta) OR (baño de bosque) OR (baignade en forêt) | 2 |
|  | AND |  |
|  | (forest therapy) OR (Relaxation Therapy) OR (Therapeutic Relaxation) OR (Relaxation, Therapeutic) OR (Therapy, Relaxation) OR (Relaxation Techniques) OR (Relaxation Technique) OR (Technique, Relaxation) OR (Techniques, Relaxation) OR (Relaxation Technics) OR (Relaxation Technic) OR (Technic, Relaxation) OR (Nature Therapy) OR (Nature Therapies) OR (Therapy, Nature) OR (Ecotherapy) OR (Ecotherapies) OR (Naturopathy) OR (Medicine, Naturopathic) OR (Naturopathic Medicine) OR (terapiadefloresta) OR (Terapia de Relaxamento) OR (Terapia por Relajación) OR (ecoterapia) OR (naturoterapia) OR (relajación terapéutica) OR (técnicas de relajación) OR (Thérapie par la relaxation) OR (Ecoterapia) OR (Relaxamento Terapêutico) OR (Terapia Apoiada pela Natureza) OR (Terapia Assitida pela Natureza) OR (Terapia da Natureza) OR (Terapia por Relaxamento) OR (Terapia por meio da Natureza) OR (Técnicas de Relaxamento) | 1.134 |
|  | Total | 1 |
| Lilacs | (Shinrin-Yoku) OR (forest bathing) OR (nature bathing) OR (banho de floresta) OR (baño de bosque) OR (baignade en forêt) | 7 |
|  | AND |  |
|  | (forest therapy) OR (Relaxation Therapy) OR (Therapeutic Relaxation) OR (Relaxation, Therapeutic) OR (Therapy, Relaxation) OR (Relaxation Techniques) OR (Relaxation Technique) OR (Technique, Relaxation) OR (Techniques, Relaxation) OR (Relaxation Technics) OR (Relaxation Technic) OR (Technic, Relaxation) OR (Nature Therapy) OR (Nature Therapies) OR (Therapy, Nature) OR (Ecotherapy) OR (Ecotherapies) OR (Naturopathy) OR (Medicine, Naturopathic) OR (Naturopathic Medicine) OR (terapiadefloresta) OR (Terapia de Relaxamento) OR (Terapia por Relajación) OR (ecoterapia) OR (naturoterapia) OR (relajación terapéutica) OR (técnicas de relajación) OR (Thérapie par la relaxation) OR (Ecoterapia) OR (Relaxamento Terapêutico) OR (Terapia Apoiada pela Natureza) OR (Terapia Assitida pela Natureza) OR (Terapia da Natureza) OR (Terapia por Relaxamento) OR (Terapia por meio da Natureza) OR (Técnicas de Relaxamento) | 3.559 |
|  | Total | 1 |
| ProQuest | (Shinrin-Yoku) OR (forest bathing) OR (nature bathing) OR (banho de floresta) OR (baño de bosque) OR (baignade en forêt) | 183 |
|  | AND |  |
|  | (forest therapy) OR (Relaxation Therapy) OR (Therapeutic Relaxation) OR (Relaxation, Therapeutic) OR (Therapy, Relaxation) OR (Relaxation Techniques) OR (Relaxation Technique) OR (Technique, Relaxation) OR (Techniques, Relaxation) OR (Relaxation Technics) OR (Relaxation Technic) OR (Technic, Relaxation) OR (Nature Therapy) OR (Nature Therapies) OR (Therapy, Nature) OR (Ecotherapy) OR (Ecotherapies) OR (Naturopathy) OR (Medicine, Naturopathic) OR (Naturopathic Medicine) OR (terapiadefloresta) OR (Terapia de Relaxamento) OR (Terapia por Relajación) OR (ecoterapia) OR (naturoterapia) OR (relajación terapéutica) OR (técnicas de relajación) OR (Thérapie par la relaxation) OR (Ecoterapia) OR (Relaxamento Terapêutico) OR (Terapia Apoiada pela Natureza) OR (Terapia Assitida pela Natureza) OR (Terapia da Natureza) OR (Terapia por Relaxamento) OR (Terapia por meio da Natureza) OR (Técnicas de Relaxamento) | 61.000 |
|  | Total | 46 |

Source: Authors' elaboration

**Table 2.** List of excluded studies after reading the full text.

| **It is not RCT or and observational studies (cohort, case control and before and after)**  **(n = 5)** |
| --- |
| Gawrych M, Słonka R. Therapeutic mountain hiking in psychiatric rehabilitation. Psychiatria i Psychologia Kliniczna. 2021;21(1):65-70. doi:10.15557/pipk.2021.0007 |
| Cho KS, Lim YR, Lee K, Lee J, Lee JH, Lee IS. Terpenes from Forests and Human Health. Toxicol Res. 2017 Apr;33(2):97-106. doi: 10.5487/TR.2017.33.2.097. Epub 2017 Apr 15. PMID: 28443180; PMCID: PMC5402865. |
| Zhang Z, Ye B. Forest Therapy in Germany, Japan, and China: Proposal, Development Status, and Future Prospects. Forests. 2022; 13(8):1289. https://doi.org/10.3390/f13081289 |
| Fan X. Policy evolution and enlightenment of Japan's forest healthcare industry. MATEC Web Conf. 2024;395:01079. doi:10.1051/matecconf/202439501079 |
| Lee, Jh., Park, Js. & Choi, S. Environmental influence in the forested area toward human health: incorporating the ecological environment into art psychotherapy. J. Mt. Sci. 17, 992–1000 (2020). https://doi.org/10.1007/s11629-019-5774-3 |
| **Does not assess efficacy (n = 2)** |
| Antonelli M, Donelli D, Maggini V, Gallo E, Mascherini V, Firenzuoli F, Gavazzi G, Zabini F, Venturelli E, Margheritini G, et al. Demographic, Psychosocial, and Lifestyle-Related Characteristics of Forest Therapy Participants in Italy: A Multicenter Cross-Sectional Survey. Healthcare. 2023; 11(11):1627. https://doi.org/10.3390/healthcare11111627 |
| Park KH. Analysis of Urban Forest Healing Program Expected Values, Needs, and Preferred Components in Urban Forest Visitors with Diseases: A Pilot Survey. Int J Environ Res Public Health. 2022;19(1):513. Published 2022 Jan 4. doi:10.3390/ijerph19010513 |
| **Does not analyze circulatory problems and mental disorders (n = 2)** |
| Morita E, Kadotani H, Yamada N, Sasakabe T, Kawai S, Naito M, Tamura T, Wakai K. The Inverse Association between the Frequency of Forest Walking (Shinrin-yoku) and the Prevalence of Insomnia Symptoms in the General Japanese Population: A Japan Multi-Institutional Collaborative Cohort Daiko Study. International Journal of Environmental Research and Public Health. 2024; 21(3):350. https://doi.org/10.3390/ijerph21030350 |
| Zhou, C., Yan, L., Yu, L. et al. Effect of Short-term Forest Bathing in Urban Parks on Perceived Anxiety of Young-adults: A Pilot Study in Guiyang, Southwest China. Chin. Geogr. Sci. 29, 139–150 (2019). https://doi.org/10.1007/s11769-018-0987-x |
| **They do not have explicit statistical data (n = 2)** |
| Li Q, Kobayashi M, Kumeda S, Ochiai T, Miura T, Kagawa T, Imai M, Wang Z, Otsuka T, Kawada T. Effects of Forest Bathing on Cardiovascular and Metabolic Parameters in Middle-Aged Males. Evid Based Complement Alternat Med. 2016;2016:2587381. doi: 10.1155/2016/2587381. Epub 2016 Jul 14. PMID: 27493670; PMCID: PMC4963577. |
| Mao GX, Cao YB, Lan XG, et al. Therapeutic effect of forest bathing on human hypertension in the elderly. J Cardiol. 2012;60(6):495-502. doi:10.1016/j.jjcc.2012.08.003 |
| **Stratified analysis of components/forests (n = 2)** |
| Weng Y, Zhu Y, Ma S, Li K, Chen Q, Wang M, Dong J. Quantitative Analysis of Physiological and Psychological Impacts of Visual and Auditory Elements in Wuyishan National Park Using Eye-Tracking. Forests. 2024; 15(7):1210. https://doi.org/10.3390/f15071210 |
| An, B. Y., Wang, D., Liu, X. J., Guan, H. M., Wei, H. X., & Ren, Z. B. (2018). The effect of environmental factors in urban forests on blood pressure and heart rate in university students. Journal of Forest Research, 24(1), 27–34. https://doi.org/10.1080/13416979.2018.1540144 |
| **It is not forest bathing (n = 1)** |
| Starry O, Viray A, Park-Egan B, Terway AC, Oxendahl T and Burdsall T (2022) A Pilot Study Considering Ecoroofs as Therapeutic Landscapes. Front. Sustain. Cities 4:811306. doi: 10.3389/frsc.2022.811306 |
| **Did not just analyze forest bathing (n=1)** |
| Serrat M, Almirall M, Musté M, et al. Effectiveness of a Multicomponent Treatment for Fibromyalgia Based on Pain Neuroscience Education, Exercise Therapy, Psychological Support, and Nature Exposure (NAT-FM): A Pragmatic Randomized Controlled Trial. J Clin Med. 2020;9(10):3348. Published 2020 Oct 18. doi:10.3390/jcm9103348 |
| **Virtual forest (n=1)** |
| Hejtmánek L, Hůla M, Herrová A and Surový P (2022) Forest digital twin as a relaxation environment: A pilot study. Front. Virtual Real. 3:1033708. doi: 10.3389/frvir.2022.1033708 |
| **No before and after comparison (n=1)** |
| Song C, Ikei H, Kobayashi M, et al. Effect of forest walking on autonomic nervous system activity in middle-aged hypertensive individuals: a pilot study. Int J Environ Res Public Health. 2015;12(3):2687-2699. Published 2015 Mar 2. doi:10.3390/ijerph120302687 |

Source: Authors' elaboration

**Table 3.** Description of objectives, dependent variables, measurement instruments and study financing (n=11).

| **Author/Year** | **Objective** | **​​Dependent variables** | **Measuring tools** | **Financial support** |
| --- | --- | --- | --- | --- |
| Quan et al 2024^1^ | Evaluate the potential psychological and physiological benefits of the Forest-based Health and Wellness (FHW) program, and whether it can help to further this type of program, with the principal aim of providing poof of its efficacy to encourage the public to enter the forest, thus supporting the development of the FHW industry to improve national health. | (a) Blood pressure (systolic/diastolic) (b) Pulse rate (c) Mood states: 1. tension-anxiety; 2. depression-despondency; 3. anger-hostility; 4. fatigue; 5. confusion, and 6. vigor | (a) LKang physical examination apparatus (b) LKang physical examination apparatus (c) POMS: Profile of Mood States | Wencheng County Innovation and Entrepreneurship Seed Fund Project, China Green Carbon Sequestration Foundation, Wenzhou Carbon Sequestration Fund Project, and Zhejiang Science and Technology Program. |
| Chun et al 2023^2^ | Identify health indices that indicate long-term effects | (a) Heart rate (b) Blood pressure (systolic/diastolic) (c) Anxiety  (d) Depression (e) Six mood states: 1. tension-anxiety; 2. depression-despondency; 3. anger-hostility; 4. fatigue; 5. confusion, and 6. vigor  (f) Quality of life (g) Positive and negative emotional affects. | (a) SA6000 (MEDICORE Co., Hanam-si, South Korea) (b) EASY X800 digital apparatus (SELVAS Healthcare, Inc., Seoul, South Korea) (c) STAI-X: State-Trait Anxiety Inventory (d) BDI: Beck Depression Inventory (e) POMS  (f) EQ-5D: Quality of Life-5 Dimension (g) PANAS: Positive and Negative Affect Schedule | P&D Program for Technology of Forest Science and the Forest Service of Korea (Korean Institute for the Promotion of Forestry). |
| Kil et al 2023^3^ | Determine whether the practise of structured forest therapy influences full attention, connection with nature, mood states, and the meanings of place and responses in the physiological health of children and adolescents with mental health disorders. | (a) Full attention state and the quality of self-awareness (b) Affective connection and actual experience with nature (c) Mood states: 1. tension-anxiety; 2. depression-despondency; 3. anger-hostility; 4. fatigue; 5. confusion, and 6. vigor (d) Blood pressure (systolic, diastolic)  (e) Pulse rate | (a) Mindfulness Attention Awareness Scale-Children (MAAS-C) (b) Connectedness to Nature Scale (CNS) (c) POMS (d) Omron 7 Series—BP652 wrist-mounted blood pressure meter (Kyoto, Japan)  (e) Omron 7 Series—BP652 wrist-mounted blood pressure meter (Kyoto, Japan) | University of Wisconsin-La Crosse |
| Bielinis et al 2021^4^ | Examine the influence of a winter forest landscape, with the ground and trees covered with snow, on the psychological relaxation of young adults | (a) Mood states: 1. tension-anxiety; 2. depression-despondency; 3. anger-hostility; 4. fatigue; 5. confusion, and 6. vigor  (b) Positive and negative emotional affects (c) Restorative effect of each environment (d) Vitality | (a) POMS (b) PANAS  (c) ROS: Restorative Outcome Scale (d) SVS: Subjective Vitality Scale | Not recorded |
| Janeczko et al 2020^5^ | Examine the restorative/renewing effects of short walks in Kabaty and Sobieski forests and an urban area. | (a) Blood pressure (systolic and diastolic)  (b) Pulse rate (c) Positive and negative emotional affects d) Restorative effect of each environment e) Vitality f) Six mood states: 1. tension-anxiety; 2. depression-despondency; 3. anger-hostility; 4. fatigue; 5. confusion, and 6. vigor | (a) Microlife BP A1 Basic blood pressure monitor (b) Microlife BP A1 Basic blood pressure monitor (c) PANAS  (d) ROS  (e) SVS;  (f) POMS | No financing |
| Bielinis et al 2019^6^ | Test the efficacy of forest therapy, and whether it has a positive effect on mood and anxiety | 1. Mood states: 1. tension-anxiety; 2. depression-despondency; 3. anger-hostility; 4. fatigue; 5. confusion, and 6. vigor 2. Anxiety level | (a) POMS (b) State-Trait Anxiety Inventory (STAI) | Faculty of Forestry at the University of Life Sciences in Poznań. |
| Kobayashi et al 2019^7^ | Determine the combined effects of walking and the environment comparing pre- and post-walk data | (a) Cortisol levels | (a) Salivette system (no. 51.1534; Sarstedt, Nümbrecht, Germany) | University of Tokyo |
| Bielinis et al 2018^8^ | Validate the effect of short winter forest baths for the psychological wellbeing, as well as the emotional, restorative, and vitalizing effects in young Polish adults, and clarify differences between the sexes | (a) Positive and negative emotional affects (b) Mood states: 1. tension-anxiety; 2. depression-despondency; 3. anger-hostility; 4. fatigue; 5. confusion, and 6. vigor  (c) restorative effect of each environment (d) Vitality | 1. PANAS 2. POMS 3. ROS 4. SVS | No financing |
| Ochiai et al 2015^9^ | Evaluate the physiological and psychological effects of a program of forest therapy in middle-aged women | (a) Pulse rate (b) Salivary cortisol-stress (c) Subjective sentiments: “comfortable to uncomfortable”, “relaxed to awake”, and “natural to artificial”  (d) Three mood states: 1. tension-anxiety; 4. fatigue, and 6. vigor | (a) HEM-1020, Omron portable digital blood pressure meter (Kyoto, Japan) (b) SalivaBio LLC No.61/524.096 saliva collector (California, United States) for the collection of samples to measure endocrine activity (c) Psychological indices derived from the Diferencial Semantic (DS) technique (d) POMS | Vehicle Racing Commemorative Foundation |
| Lee et al 2011^10^ | Provide scientific evidence that supports the efficacy of forest bathing as a natural therapy, investigating its physiological benefits using biological indicators in external environments. | (a) Heart rate (b) Cortisol levels (c) Blood pressure (systolic and diastolic) (d) Subjective sentiments: comfortable, tranquil, and reinvigorated. (e) Six mood states: 1. tension-anxiety; 2. depression-despondency; 3. anger-hostility; 4. fatigue; 5. confusion, and 6. vigor | (a) Activtracer AC-301A portable electrocariograph (GMS, Japan) (b) Salivette (no. 51,1534) for the collection of saliva samples (Sarstedt, Alemanha). (c) HEM-1000 Omron portable blood pressure monitor (Tokyo, Japan). (d) Diferencial Semantic (DS) techniques  (e) POMS | Japanese Ministry of Education, Culture, Sports, Science, and Technology (MEXT) |
| Morita et al 2007^11^ | (1) examine the acute psychological effects of shinrin-yoku in a large number of participants; and (2) identify the factors related to these effects. | (a) Level of anxiety | (a) STAI-S: State Trait Anxiety Inventory A-State Scale | Tokyo University Forest; Forestry and Forest Products Research Institute; and the Society for the Study of Forest Therapy, Japan |

Source: Authors' elaboration

**Table 4.** Assessment of outcomes evidenced in the systematic review using the GRADE pro tool.

| **Certainty assessment** | | | | | | | **Certainty** |
| --- | --- | --- | --- | --- | --- | --- | --- |
| **№ of studies** | **Study design** | **Risk of bias** | **Inconsistency** | **Indirectness** | **Imprecision** | **Other considerations** |  |
| 5 | non-randomised studies | serious^a^ | serious^b^ | not serious | not serious | publication bias strongly suspected all plausible residual confounding would reduce the demonstrated effect^c^ | ⨁◯◯◯ Very low^a,b,c^ |
| 3 | non-randomised studies  (2 studies)  randomised studies  (1 study) | serious^a^ | serious^b^ | not serious | not serious | publication bias strongly suspected all plausible residual confounding would reduce the demonstrated effect^c^ | ⨁◯◯◯ Very low^a,b,c^ |
| 8 | non-randomised studies | serious^a^ | serious^b^ | not serious | not serious | publication bias strongly suspected all plausible residual confounding would reduce the demonstrated effect^c^ | ⨁◯◯◯ Very low^a,b,c^ |
| 7 | non-randomised studies | serious^a^ | serious^b^ | not serious | not serious | publication bias strongly suspected all plausible residual confounding would reduce the demonstrated effect^c^ | ⨁◯◯◯ Very low^a,b,c^ |
| 7 | non-randomised studies | serious^a^ | serious^b^ | not serious | not serious | publication bias strongly suspected all plausible residual confounding would reduce the demonstrated effect^c^ | ⨁◯◯◯ Very low^a,b,c^ |
| 7 | non-randomised studies | serious^a^ | serious^b^ | not serious | not serious | publication bias strongly suspected all plausible residual confounding would reduce the demonstrated effect^c^ | ⨁◯◯◯ Very low^a,b,c^ |
| 7 | non-randomised studies | serious^a^ | serious^b^ | not serious | not serious | publication bias strongly suspected all plausible residual confounding would reduce the demonstrated effect^c^ | ⨁◯◯◯ Very low^a,b,c^ |
| 7 | non-randomised studies | serious^a^ | serious^b^ | not serious | not serious | publication bias strongly suspected all plausible residual confounding would reduce the demonstrated effect^c^ | ⨁◯◯◯ Very low^a,b,c^ |
| 3 | non-randomised studies | serious^a^ | serious^b^ | not serious | not serious | publication bias strongly suspected all plausible residual confounding would reduce the demonstrated effect^c^ | ⨁◯◯◯ Very low^a,b,c^ |
| 3 | non-randomised studies | serious^a^ | serious^b^ | not serious | not serious | publication bias strongly suspected all plausible residual confounding would reduce the demonstrated effect^c^ | ⨁◯◯◯ Very low^a,b,c^ |
| 3 | non-randomised studies | serious^a^ | serious^b^ | not serious | not serious | publication bias strongly suspected all plausible residual confounding would reduce the demonstrated effect^c^ | ⨁◯◯◯ Very low^a,b,c^ |
| 3 | non-randomised studies | serious^a^ | serious^b^ | not serious | not serious | publication bias strongly suspected all plausible residual confounding would reduce the demonstrated effect^c^ | ⨁◯◯◯ Very low^a,b,c^ |

**CI:** confidence interval; **MD:** mean difference

Source: Authors' elaboration

#### Explanations

a. Absence of masking (blinding)- Bielinis et al 2018; Bielinis et al 2019; Bielinis et al 2021; Chun et al 2023; Janeczko et al 2020; kil et al 2023; Kobayashi et al 2019; Lee et al 2011; Morita et al 2007; Ochiai et al 2015; e Quan et al 2024.

b. Heterogeneity (Population): Ochiai et al 2015 was only done with women; kil et al 2023 was only done with children and adolescents; Kobayashi et al 2019 was only done with men; Bielinis et al 2019 and Morita et al 2007 were with adults over 40 years old.

c. Confounding factors: Winter temperature (Bielinis et al 2018, and Bielinis et al 2021); Walking has not been isolated (Bielinis et al 2018; Bielinis et al 2019; Bielinis et al 2021; Chun et al 2023; Janeczko et al 2020; kil et al 2023; Kobayashi et al 2019; Morita et al 2007 ;Ochiai et al 2015; Quan et al 2024) and Observation in the forest in the form of standing, sitting or lying down (Bielinis et al 2021).

**
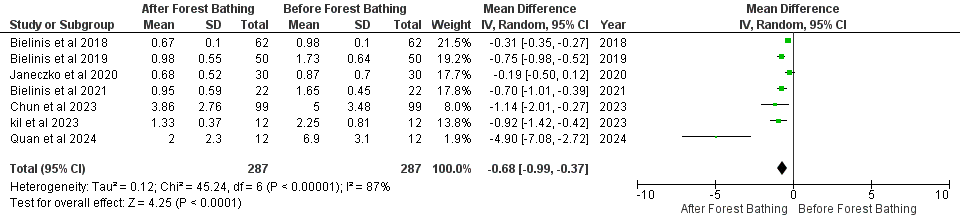
**

**Figure 1.** Meta-analyses of confusion for psychological outcomes related to mental disorders.

Source: Authors' elaboration


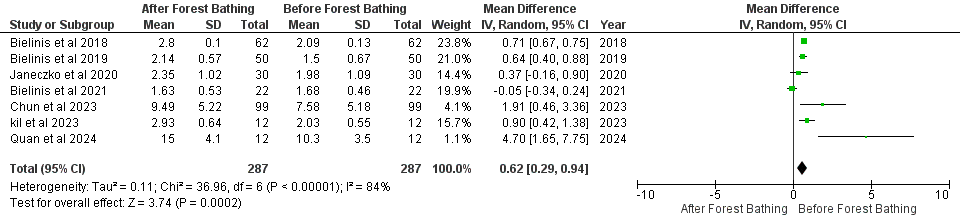


**Figure 2.** Meta-analyses of vigor for psychological outcomes related to mental disorders.

Source: Authors' elaboration

**
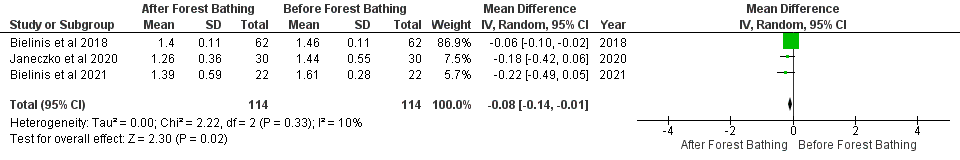
**

**Figure 3.** Meta-analyses of negative emotional affect for psychological outcomes related to mental disorders. *This meta-analysis considered the non-diseased subgroup.

Source: Authors' elaboration


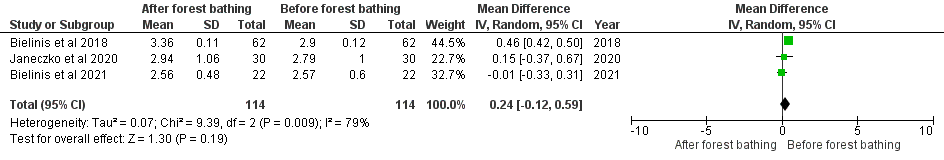


**Figure 4.** Meta-analyses of positive emotional affect for psychological outcomes related to mental disorders. *This meta-analysis considered the non-diseased subgroup.

Source: Authors' elaboration


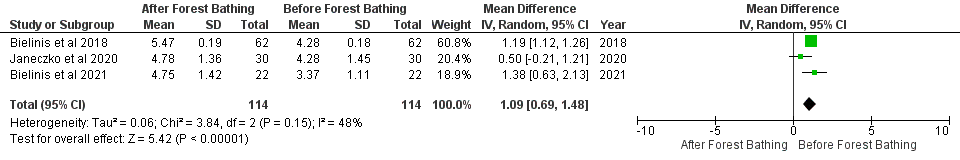


**Figure 5.** Meta-analyses of restorative effect of each environment for psychological outcomes related to mental disorders. *This meta-analysis considered the non-diseased subgroup.

Source: Authors' elaboration


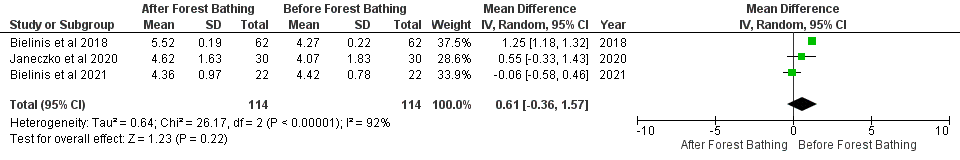


**Figure 6.** Meta-analyses of vitality for psychological outcomes related to mental disorders.

Source: Authors' elaboration*This meta-analysis considered the non-diseased subgroup.


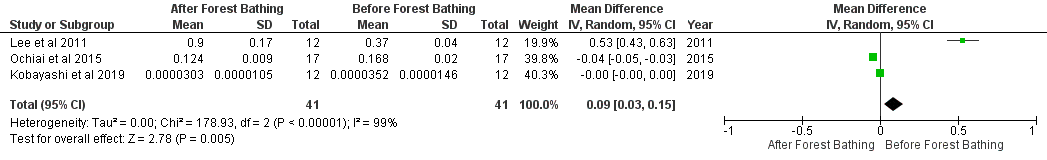


**Figure 7.** Meta-analyses of salivary cortisol for psychological outcomes related to mental disorders.

Source: Authors' elaboration


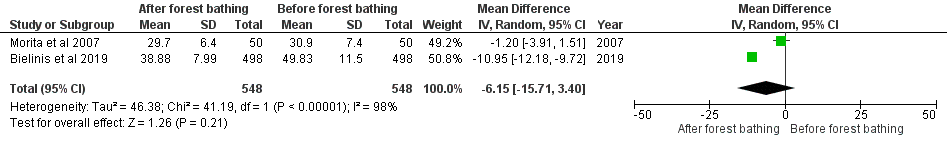


**Figure 8.** Meta-analyses of anxiety for psychological outcomes related to mental disorders.

Source: Authors' elaboration*This meta-analysis considered the non-diseased subgroup.


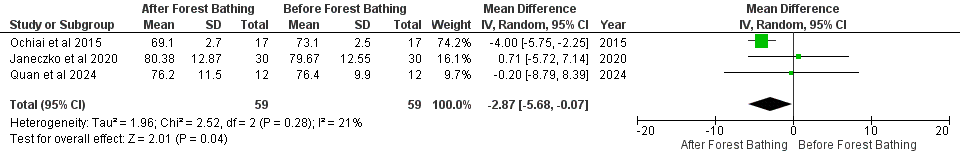


**Figure 9.** Subgroup meta-analyses for non-diseased people of pulse rate for physiological outcomes related to circulatory disorders.

Source: Authors' elaboration


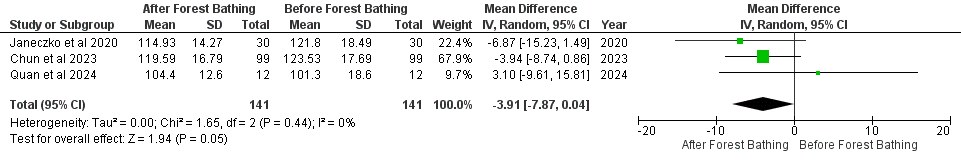


**Figure 10.** Subgroup meta-analyses for non-diseased people of systolic blood pressure for physiological outcomes related to circulatory disorders.

Source: Authors' elaboration


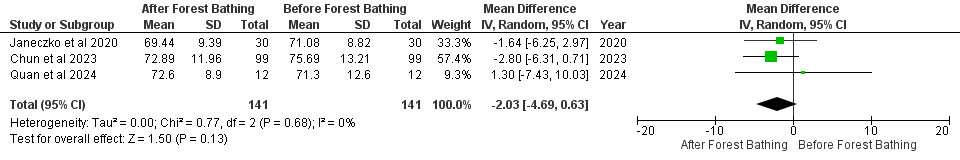


**Figure 11.** Subgroup meta-analyses for non-diseased people of diastolic blood pressure for physiological outcomes related to circulatory disorders.

Source: Authors' elaboration


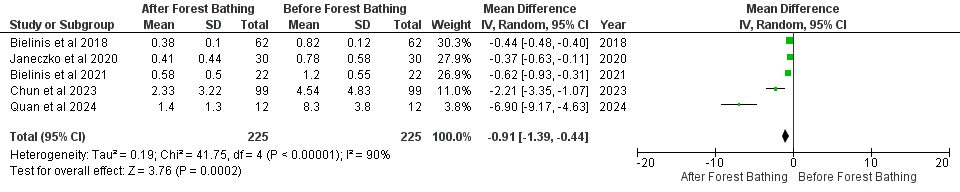


**Figure 12.** Subgroup meta-analyses for non-diseased people of tension-anxiety for psychological outcomes related to mental disorders.

Source: Authors' elaboration


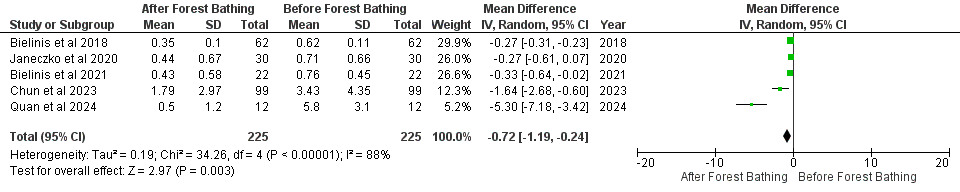


**Figure 13.** Subgroup meta-analyses for non-diseased people of depression-discouragement for psychological outcomes related to mental disorders.

Source: Authors' elaboration


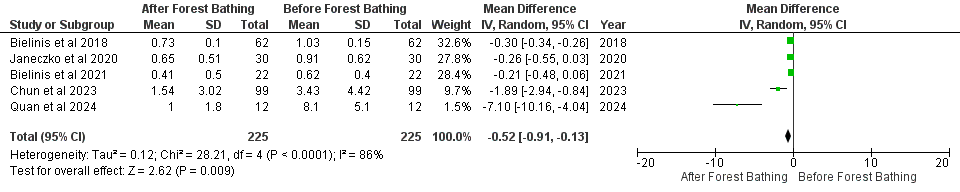


**Figure 14.** Subgroup meta-analyses for non-diseased people of anger-hostility for psychological outcomes related to mental disorders.

Source: Authors' elaboration


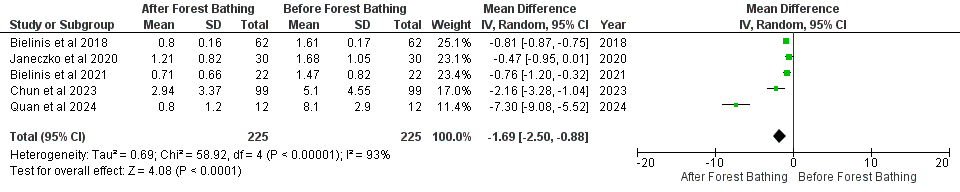


**Figure 15.** Subgroup meta-analyses for non-diseased people of fatigue for psychological outcomes related to mental disorders.

Source: Authors' elaboration


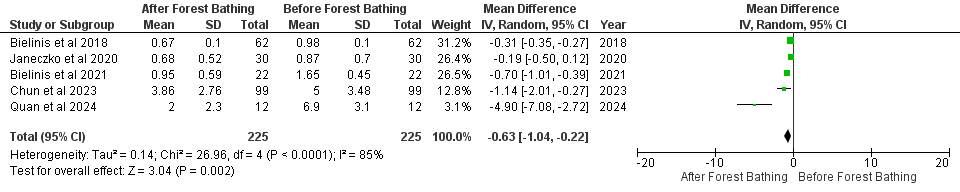


**Figure 16.** Subgroup meta-analyses for non-diseased people of confusion for psychological outcomes related to mental disorders.

Source: Authors' elaboration


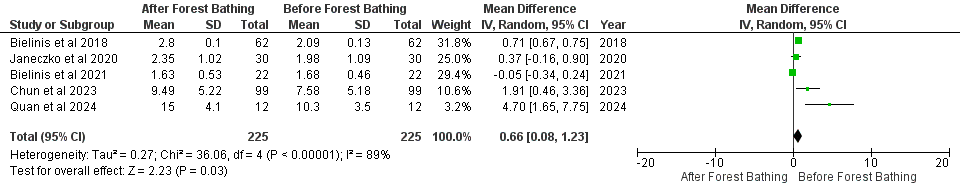


**Figure 17.** Subgroup meta-analyses for non-diseased people of vigor for psychological outcomes related to mental disorders.

Source: Authors' elaboration


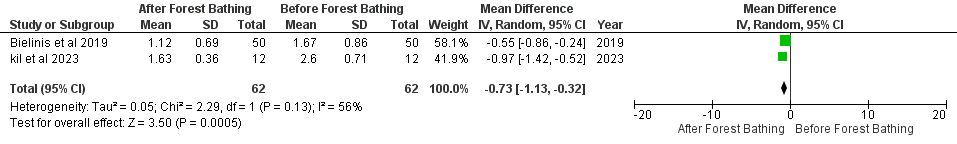


**Figure 18.** Subgroup meta-analyses for diseased people of tension-anxiety for psychological outcomes related to mental disorders.

Source: Authors' elaboration


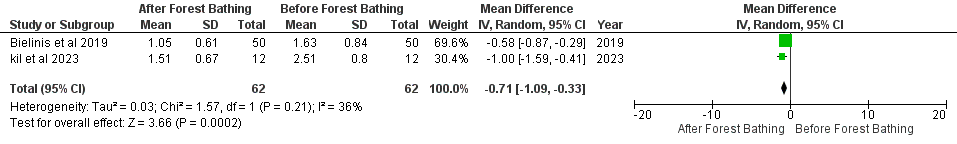


**Figure 19.** Subgroup meta-analyses for diseased people of depression-discouragement for psychological outcomes related to mental disorders.

Source: Authors' elaboration


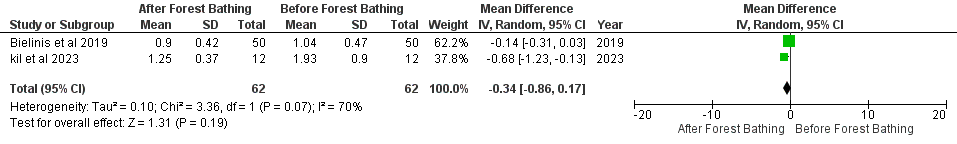


**Figure 20.** Subgroup meta-analyses for diseased people of anger-hostility for psychological outcomes related to mental disorders.

Source: Authors' elaboration


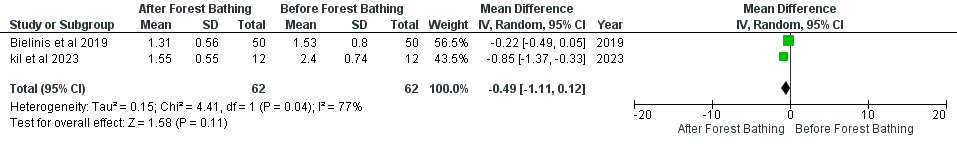


**Figure 21.** Subgroup meta-analyses for diseased people of fatigue for psychological outcomes related to mental disorders.

Source: Authors' elaboration


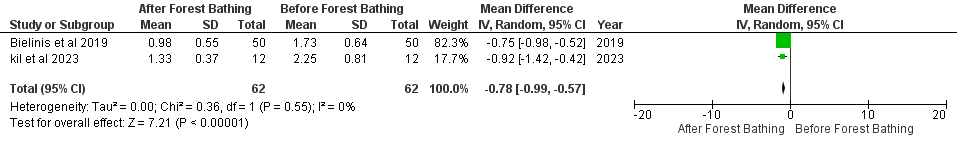


**Figure 22.** Subgroup meta-analyses for diseased people of confusion for psychological outcomes related to mental disorders.

Source: Authors' elaboration


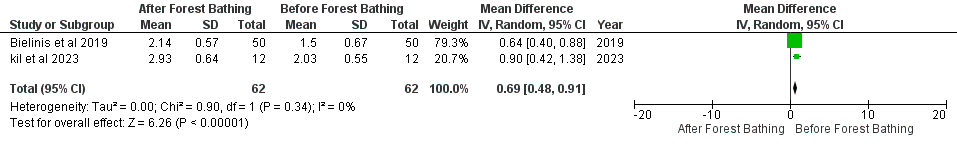


**Figure 23.** Subgroup meta-analyses for diseased people of vigor for psychological outcomes related to mental disorders.

Source: Authors' elaboration

**Referências Biliográficas**

1. Quan W, Yu S, Huang Q and Ying M (2024). The effect of forest-based health and wellness on the stress-relieve of middle-aged people. Front. Public Health 12:1366339. Doi: 10.3389/fpubh.2024.1366339
2. Chun, H.-r.; Cho, I.; Choi, Y.Y.; Park, S.; Kim, G.; Cho, S.-i. Effects of a Forest Therapy Program on Physical Health, Mental Health, and Health Behaviors. Forests 2023, 14, 2236. <https://doi.org/10.3390/f14112236>
3. Namyun Kil, Jin Gun Kim, Emily Thornton, Amy Jeranek, Psychological and Physiological Health Benefits of a Structured Forest Therapy Program for Children and Adolescents with Mental Health Disorders, International Journal of Mental Health Promotion, Volume 25, Issue 10, 2023, Pages 1117-1125. <https://doi.org/10.32604/ijmhp.2023.022981>.
4. Bielinis E, Janeczko E, Takayama N, Zawadzka A, Słupska A, Piętka S, et al. (2021) The effects of viewing a winter forest landscape with the ground and trees covered in snow on the psychological relaxation of young Finnish adults: A pilot study. PLoS ONE 16(1): e0244799. <https://doi.org/10.1371/journal.pone.0244799>
5. Janeczko E, Bielinis E, Wójcik R, Woźnicka M, Kędziora W, Łukowski A, Elsadek M, Szyc K, Janeczko K. When Urban Environment Is Restorative: The Effect of Walking in Suburbs and Forests on Psychological and Physiological Relaxation of Young Polish Adults. Forests. 2020; 11(5):591. <https://doi.org/10.3390/f11050591>
6. Bielinis E, Jaroszewska A, Łukowski A, Takayama N. The Effects of a Forest Therapy Programme on Mental Hospital Patients with Affective and Psychotic Disorders. Int J Environ Res Public Health. 2019;17(1):118. Published 2019 Dec 23. doi:10.3390/ijerph17010118
7. Kobayashi H, Song C, Ikei H, Park B-J, Kagawa T and Miyazaki Y (2019) Combined Effect of Walking and Forest Environment on Salivary Cortisol Concentration. Front. Public Health 7:376. doi: 10.3389/fpubh.2019.00376
8. Ernest Bielinis, Norimasa Takayama, Sergii Boiko, Aneta Omelan, Lidia Bielinis, The effect of winter forest bathing on psychological relaxation of young Polish adults, Urban Forestry & Urban Greening, Volume 29, 2018, Pages 276-283, <https://doi.org/10.1016/j.ufug.2017.12.006>.
9. Ochiai H, Ikei H, Song C, Kobayashi M, Miura T, Kagawa T, Li Q, Kumeda S, Imai M, Miyazaki Y. Physiological and Psychological Effects of a Forest Therapy Program on Middle-Aged Females. Int J Environ Res Public Health. 2015 Dec 1;12(12):15222-32. doi: 10.3390/ijerph121214984.
10. Lee J, Park BJ, Park BJ, et al. Effect of forest bathing on physiological and psychological responses in young Japanese male subjects. Public Health. 2011 Feb;125(2):93-100. DOI: 10.1016/j.puhe.2010.09.005.
11. Morita E, Fukuda S, Nagano J, et al. Psychological effects of forest environments on healthy adults: Shinrin-yoku (forest-air bathing, walking) as a possible method of stress reduction. Public Health. 2007;121(1):54-63. doi:10.1016/j.puhe.2006.05.024
